# Population Dynamics in Italian Canids between the Late Pleistocene and Bronze Age

**DOI:** 10.3390/genes11121409

**Published:** 2020-11-26

**Authors:** Kyriaki Koupadi, Francesco Fontani, Marta Maria Ciucani, Elena Maini, Sara De Fanti, Maurizio Cattani, Antonio Curci, Gabriele Nenzioni, Paolo Reggiani, Adam J. Andrews, Stefania Sarno, Carla Bini, Susi Pelotti, Romolo Caniglia, Donata Luiselli, Elisabetta Cilli

**Affiliations:** 1 Hellenic Ministry of Culture and Sports, Ephorate of Antiquities of the City of Athens, Makriyianni 2-4, 11742 Athens, Greece; koupadikrk@gmail.com; 2 Department of Cultural Heritage, University of Bologna, Via Degli Ariani 1, 48121 Ravenna, Italy; francesco.fontani@studio.unibo.it (F.F.); a.andrews@unibo.it (A.J.A.); donata.luiselli@unibo.it (D.L.); 3 Section for Evolutionary Genomics, the GLOBE Institute, University of Copenhagen, Oester Voldgade 5-7, 1350 Copenhagen, Denmark; martamariaciucani@gmail.com; 4 ArcheoLaBio—Research Centre for Bioarchaeology, Department of History and Cultures, University of Bologna, Via San Vitale 30, 48121 Ravenna, Italy; elena.maini@unibo.it (E.M.); antonio.curci@unibo.it (A.C.); 5 Department of Biological Geological and Environmental Sciences, University of Bologna, via Selmi 3, 40126 Bologna, Italy; sara.defanti2@unibo.it (S.D.F.); stefania.sarno2@unibo.it (S.S.); 6 Interdepartmental Centre “Alma Mater Research Institute on Global Challenges and Climate Change (Alma Climate)”, University of Bologna, Via Petroni 26, 40126 Bologna, Italy; 7 Department of History and Cultures, University of Bologna, Via San Vitale 30, 48121 Ravenna, Italy; maurizio.cattani@unibo.it; 8 Museo della Preistoria “Luigi Donini”, Via Fratelli Canova 49, 40068 San Lazzaro di Savena, BO, Italy; gabriele.nenzioni@comune.sanlazzaro.bo.it; 9 Paleostudy, Via Martiri delle Foibe 1, 35028 Piove di Sacco, PD, Italy; paleostudy@libero.it; 10 Department of Medical and Surgical Sciences, University of Bologna, Via Irnerio 49, 40126 Bologna, Italy; carla.bini@unibo.it (C.B.); susi.pelotti@unibo.it (S.P.); 11 Unit for Conservation Genetics (BIO-CGE), Italian Institute for Environmental Protection and Research (ISPRA), Via Ca’ Fornacetta 9, 40064 Ozzano dell’Emilia, BO, Italy; romolo.caniglia@isprambiente.it

**Keywords:** ancient DNA, dogs, domestication, mitochondrial DNA, population genetics, archaeology, Italy

## Abstract

Dog domestication is still largely unresolved due to time-gaps in the sampling of regions. Ancient Italian canids are particularly understudied, currently represented by only a few specimens. In the present study, we sampled 27 canid remains from Northern Italy dated between the Late Pleistocene and Bronze Age to assess their genetic variability, and thus add context to dog domestication dynamics. They were targeted at four DNA fragments of the hypervariable region 1 of mitochondrial DNA. A total of 11 samples had good DNA preservation and were used for phylogenetic analyses. The dog samples were assigned to dog haplogroups A, C and D, and a Late Pleistocene wolf was set into wolf haplogroup 2. We present our data in the landscape of ancient and modern dog genetic variability, with a particular focus on the ancient Italian samples published thus far. Our results suggest there is high genetic variability within ancient Italian canids, where close relationships were evident between both a ~24,700 years old Italian canid, and Iberian and Bulgarian ancient dogs. These findings emphasize that disentangling dog domestication dynamics benefits from the analysis of specimens from Southern European regions.

## 1. Introduction

Domestication processes involving plants and animals have had a strong biological and cultural impact on humans. Dog domestication in particular is one of the most fascinating and unresolved processes. Our special bond with “man’s best friend” traces back its roots to the Late Pleistocene [1]. Thus, dogs were the first species domesticated, originating from an unknown population of grey wolves [2,3,4,5], and predated the appearance of agriculture in the archeological record by several thousand years [2].

The origin of dogs dates to the ancient coevolution and mutualism established between Paleolithic humans and wolves [3,6,7,8]. However, details such as the time and place of dog domestication, the motivation behind this process, and how it occurred are yet to be clarified [2,6,9,10]. Uncertainty regarding dog domestication is mainly due to a lack of available data, and those obtained by key morphological characters that were not fixed during the initial phases of the domestication process. Disentangling dog genetics is complicated due to: (i) the occurrence of several population bottlenecks in ancient wolves, most notably 20,000 years ago [11], along with the megafauna “crisis” during the Last Glacial Maximum (LGM), and because of human persecution towards wolves in the last 200 years, which dramatically reduced their genetic variability [12,13]; (ii) the explosive radiation of dog breeds in the 19th century, with more than 400 officially recognized breeds today; and (iii) recurrent post-divergence dog-to-wolf gene flow events. The combination of these factors makes the phylogeographic history of wolves and dogs difficult to reconstruct based on either archaeological data or contemporary genetic patterns from wolves and dogs. Here, ancient DNA offers utility towards reconstructing past genetic variability, and the tracking of evolutionary patterns and population dynamics through time.

Archaeological and morphological records of ancient canid remains from France, Germany (Bonn-Oberkassel) and Spain indicate that the first confidently identified domesticated dogs originated by at least 15,000 or 13,500 years ago [14,15,16,17,18,19,20]. In comparison, East Asia returned its earliest archaeological dog remains dated to 12,500 years ago [2]. Some studies highlighted Paleolithic dogs dated between 40,000 and 16,000 before present (BP) from Goyet Cave, Belgium [21], Russia [22,23,24,25], Germany [26] and the Czech Republic [27,28], but the taxonomy of these proposed Paleolithic dogs is still contentious. Paleogenomic analysis is yet to unambiguously solve this debate as the mitogenomes of the ancient Paleolithic dogs analyzed by Thalmann et al. [29] constitute an ancient sister group to all modern dogs and wolves.

Recent advances in DNA sequencing technologies and protocols on ancient biological remains have enabled the production of data at unprecedented resolution. Paleogenomic studies attested a long-term and close history of dogs alongside humans during the Paleolithic [5,9,10], supporting predictions based on the genome of a 35,000-year-old wolf Taimyr fossil [30]. In particular, Bergstrom and colleagues [5] detected a deep diversification of dogs at the onset of the Holocene, with the presence of at least five dog ancestry lineages 11,000 years ago, testifying a deep genomic history of dogs during the Paleolithic. By comparing ancient human and dog datasets, they also inferred dispersal patterns, showing interesting concordance (Levant-related ancestry in Africa and early agricultural Europe) or discrepancy (steppe pastoralist expansion in Eurasia) between population dynamics of these two species during time and space [5]. The genomic data suggest a single [5,10] or dual [9] origin of domestic dogs. However, the acquisition of a broader set of ancient samples is required to further clarify the details of the dog domestication process.

Especially in Europe, there is a limited availability of data on the genetic diversity of wolves prior to their domestication and their contribution to the genomic diversity of dogs [11,13,31,32,33]. In addition, genetic data on early dogs is lacking for most areas of Europe [9,10], particularly in the southern regions. To this end, the Italian peninsula, due to its crucial geographic position as important glacial refugium during the LGM (circa 24,000–18,000 years BP), is particularly interesting in the study of speciation, population dynamics and migration during this relevant period in the dog domestication timeline.

Few ancient Italian canid samples have been genetically analyzed at present, and these were predominantly wolves [12,13,31,34,35]. A recent study showed the presence of a haplotype belonging to the dog haplogroup (Hg) A in two canids from Italy dated between ~24,700 and ~20,000 years BP [13]. Those results, combined with the findings of Pires et al. [33] on the occurrence of Late Pleistocene wolves and Mesolithic dog carrying a Hg A canine haplotype in another glacial refugium, the Iberian Peninsula, stimulate interest towards these areas and their role in the dog domestication issue as a whole. Neolithic Bulgarian dogs in the Balkan Peninsula [6] with high percentages of Hg A and B support the findings of Pires et al. [33]. These results are of particular interest because, according to previous data [36], Hg A-dogs were only estimated to have arrived in Europe during the Bronze Age. Therefore, this reiterates the need to broaden investigations to these areas.

In this study, we provide a preliminary overview of the ancient Italian mitochondrial DNA (mtDNA) variability of dogs belonging to a large temporal transect, spanning from the Late Pleistocene to the Bronze Age. We collected ancient specimens from Northern Italy, which were analyzed at a portion of the hypervariable region 1 (HVR-1) of the mitochondrial DNA, which is highly informative for wolf and dog phylogenetics. We contextualized the data alongside a comprehensive panel of ancient and modern dogs and wolves. In particular, this study aims to: (1) investigate the genetic variability of ancient Italian dogs; and (2) improve the data about dog haplogroups dynamics in Southern Europe, especially regarding Hg A.

## 2. Materials and Methods

### 2.1. Sample Selection

**Figure 1:**
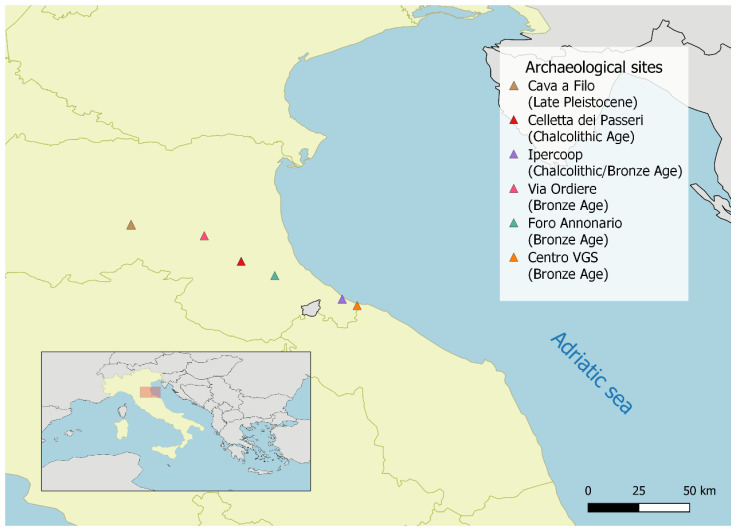
Map of the archaeological excavation locations from which the selected specimens were collected. Map created using QGIS [35].

We collected 27 specimens (Table 1, Figure S1 and Table S1) from different archaeological sites and rescue excavations in Italy (Figure 1). Sampling was mainly focused on Northeast Italy, where it was possible to recover specimens spanning a large timeframe (~25,000–3100 years BP). The majority of samples were found in excavation layers that were estimated to belong to the Bronze Age, except from three sites. These were: a phalanx (CAF18.0066) from Cava a Filo, dated at ~24,400 years BP, a tooth (NIP10) from Celletta dei Passeri, in Forlì, dated to the Chalcolithic period, and four specimens (IPC1, IPC6, IPC7, IPC8) from Riccione, dated to the Chalcolithic to Bronze Age transition. Further details about the archaeological sites are available in File S1. Information about samples can be retrieved from Table 1 and Table S1.

Despite that this study was mainly focused on the genetic origin and dynamics of dogs in Italy, two samples identified as wolf (CVGS3 and CAF18.0066) were added to genetically characterize the former population of wolves, and because previous findings highlighted interesting results about Late Pleistocene dog haplogroup dynamics in this area [13].

Samples were selected by taking into consideration the stratigraphic unit where they were found, the type, and the preservation state of the remains available. Due to the occurrence of mixed or incomplete remains, we selected specimens from different stratigraphic units to improve the likelihood of studying different individuals. We preferentially selected skeletal elements which allow for a better endogenous DNA preservation, such as the petrous bones [37,38] and teeth, especially those preserved inside the alveolus and thus less prone to contamination [39,40].

### 2.2. Experimental Procedure

Genetic analysis took place in the ancient DNA laboratory (aDNALab) of the University of Bologna, in Ravenna, exclusively dedicated to the treatment of ancient DNA following high sterility standards. Post-PCR procedures took place in a separate laboratory. Strict ancient DNA authenticity criteria were followed during the analyses [41,42,43,44]. The aDNALab is organized in separate rooms, each dedicated to different experimental procedures (sampling, DNA extraction and amplification sample set up). All materials and instruments used were DNA free and sterilized before use using bleach and/or UV light, depending on their composition. Negative controls were prepared alongside samples to confirm the absence of exogenous DNA and investigate the presence of intra-laboratory contamination.

The specimens were photographed and then decontaminated, i.e., they were exposed to UV light (~250 nm) and their surface was mechanically cleaned before sampling. The majority of samples extracted were obtained in powder, by means of a dental drill with sterile drill bits or diamond cutting discs, using different approaches based on the type of specimen. Teeth were either drilled directly from the root or carefully ground after the separation of the root from the crown. In some cases, in which this separation was not feasible, the whole tooth was pulverized. Samples CAF18.0066, NIP10, IPC6, IPC7 and IPC8 were processed in pieces [45]. All samples were extracted and amplified twice to confirm the data.

### 2.3. DNA Isolation

A two-day extraction protocol, slightly modified from those in the literature [45,46], was followed. The samples were extracted in groups of four or five to avoid inter-contamination, using a maximum of 300 mg of material. Samples CAF18.0066, NIP10, IPC6, IPC7 and IPC8 underwent a pre-extraction step to minimize the presence of any exogenous DNA in the sample extracts [45]. This step included a partial digestion in 3 mL extraction buffer (0.45 M EDTA, 0.25 mg/mL Proteinase K) for 20 min at 37 °C under constant rotation. Samples were then centrifuged, and the pre-digest supernatants were removed. At this point, all samples underwent the same extraction protocol, by adding another 3 mL extraction buffer. The samples were incubated overnight under constant rotation at 37 °C. Binding and subsequent phases were performed using the MinElute PCR Purification Kit (Qiagen, Hilden, Germany) and reservoirs, as suggested by Dabney et al. [46]. DNA was eluted in 50 μL of TET buffer (10 mM Tris-HCl, 1 mM EDTA, 0.05% Tween-20).

### 2.4. DNA Amplification and Sequencing

Extracts were amplified using polymerase chain reaction (PCR). Four different pairs of primers were used, targeting the hypervariable region 1 (HVR-1) of the mitochondrial genome. Due to the highly fragmented and fragile nature of ancient DNA, three overlapping amplicons were targeted using three sets of primers previously tested by Ersmark and colleagues, to obtain a 360 bp fragment [47]. The first fragment fell between positions 15,411–15,558 (MS_wolf1 and dogdl5, length 148 bp), the second between 15,481–15,684 (MS_Wolf2F and MS_Wolf2R, length 205 bp) and the third between 15,602–15,812 (MS_Wolf3F and MS_Wolf3R, length 211 bp). Since several specimens failed to amplify due to degradation, primers targeting a short fragment (length 99 bp, 57 bp without primers), located between 15,588–15,687 (HVR1-wolf-F and -R), were used [48]. The 57 bp fragment, despite its limited size, includes 30 polymorphic sites that represent the majority of the informative nucleotide positions of the mtDNA control region, already tested for phylogenetic purposes in several studies concerning ancient canids [21,32,47,48].

All PCR reactions contained 5–10 ng DNA extract in a total reaction volume of 25 μL. The thermal cycler was set at 94 °C for denaturation, and 72 °C for elongation for all reactions. We used three different temperatures for annealing depending on the couples of primers used (55 °C for fragment 1, 53 °C for fragments 2 and 3 and 50 °C for the short fragment). The number of cycles varied between 40 and 50. Amplification success was determined by gel electrophoresis.

Prior to sequencing, samples were purified using ExoSAP PCR Product Cleanup Reagent (ThermoFisher, Waltham, MA, USA), following the manufacturer’s instructions.

The sequencing reactions were prepared in the Laboratory of Molecular Anthropology & Centre for Genome Biology of the Department of Biological, Geological and Environmental Sciences (BiGeA, Bologna, Italy), using the BigDye Terminator v1.1 Cycle Sequencing Kit (Thermo Fisher Scientific, Waltham, MA, USA). PCR products were separated through CE using a POP1 polymer on SeqStudio Genetic Analyzer (Applied Biosystems, Foster City, CA, USA) and analyzed by the Sequencing Analysis Software v.7 (Applied Biosystems) in the Forensic Genetics Laboratory of the University of Bologna. The samples were sequenced in both directions.

Sequences are available on GenBank (www.ncbi.nlm.nih.gov/genbank; accession numbers: MW092060-MW092070, see Table S1).

### 2.5. Haplotype Identification and Phylogenetic Analyses

Electropherograms were analyzed and edited using the software FinchTV (Geospiza Inc., Seattle, WA, USA) and BioEdit [49,50]. Sequences were aligned using the dog mtDNA sequences NC_002008 and EU789787 as reference, following the steps of previously published papers [6,9,51,52]. Haplogroup assignments were determined through a phylogenetic analysis by creating UPGMA trees in MEGA X (Penn State University, PA, USA) [53], using reference samples with well-defined dog matrilines, available from previous studies [54,55]. The haplogroup of the ancient samples analyzed was also tested through the MitoToolPy program [56], with reference sequence EU789787 [57].

In order to investigate haplogroup patterns and dynamics, ggplot2 [58], a data visualization package in R [59], was used to create a graphical representation of the proportions of haplogroup through time. The plot included the ancient Eurasian dogs from this study and those available in the literature [6,9,10,13,29,31,33,34,52,60,61,62,63,64] (Table S1).

With the aim to reconstruct the ancient genetic variability of our samples and the Italian specimens sequenced so far [13,31], these sequences were checked against the GenBank database [65]. Thus, we included PIC1, PIC2, PIC3, PIC4 and PIC5 samples from Verginelli et al. [31] and also OWW4 and OWW9 samples from Ciucani et al. [13]. We added Italian samples from previous studies because recently, several papers were published about Late Pleistocene ancient wolves [66], historical sledge dogs [67], ancient wolves and dogs from Iberia [33] and ancient Bulgarian dogs [6].

A database of published mtDNA sequences (Table S1) was constructed to set the data produced herein in the wider context of ancient and modern samples. The ancient database used contains ancient samples ranging from the Late Pleistocene to Late Antiquity, published by previous studies [6,9,13,29,31,33,34,36,52,57,62,63,64,68,69,70,71,72,73,74,75,76]. In particular, two alignments of different lengths, 57 bp and 360 bp, were prepared to match the lengths of the sequences obtained herein, and those Italian canid remains previously analyzed [13,31,34]. These alignments contain 1731 and 134 sequences, respectively.

Collapsing of the sequences into haplotypes was done using the software DnaSP v.5.10.01 [77]. The shortest alignment (57 bp) was used to build a Median Joining network (ε = 0) through the Network software [78,79].

A Bayesian phylogenetic tree was constructed with the software MrBayes v. 3.2 [80] using the 360 bp alignment (Table S1) and a coyote sequence (*Canis latrans*, GenBank acc. no.: DQ480509) as outgroup. The tree was generated using the best fitting evolutionary model GTR+I+γ, 10,000,000 Markov Chain Monte Carlo (MCMC) generations, with a 10% burn-in, sampling at each 1000th interval. The α parameter was 0.801 and the proportion of invariable sites was 0.602. Potential scale reduction factors, and effective sample size parameters were interpreted as output to confirm MCMC convergence [80].

## 3. Results

Out of 27 selected specimens, 11 samples (Table 1 and Table 2) were successfully amplified and complied with the selected authentication criteria (i.e., the results were confirmed by multiple extractions and amplifications, see also the “Experimental Procedure” subsection). The materials used and the guidelines followed during the experiments provide confidence on the authenticity of the DNA fragments retrieved. The absence of amplified fragments in the negative controls, and the length and preservation of the obtained sequences validated our methodology to prevent and detect contamination. The successful samples belonged to the archaeological sites of Cava a Filo, Ipercoop Riccione, via Ordiere Solarolo, and Foro Annonario Cesena.

### 3.1. Haplotype Variability

The 11 sequences were assigned to seven different haplotypes. One sequence (CAF18.0066) was assigned to the wolf haplogroup 2, based on the subdivision of two main and distinct wolf mitochondrial haplogroups (known as Hg 1 and Hg 2) proposed by Pilot et al. [32]. The remaining sequences were assigned to the established dogs haplogroups A–D [54,68] though MitoToolPy, whose results were confirmed by their positions in the two phylogenetic trees (Figures S2 and S3). Among the 10 dog haplotypes, five sequences were assigned to dog haplogroup A, four to dog haplogroup C and one to haplogroup D (Table 2). The Table S1 provides an overview of the variants of the samples here analyzed, with respect to the reference (EU789787).

**Figure 2:**
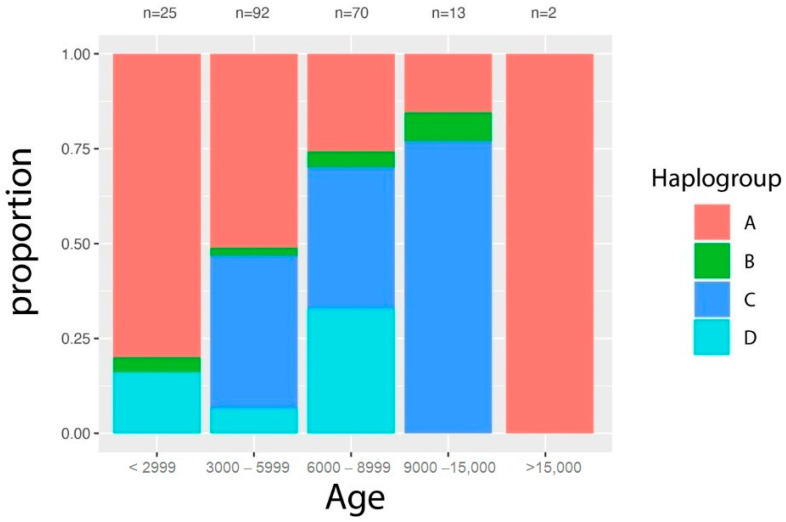
Frequency of the four main mtDNA haplogroups (A–D) in Eurasia in different time periods. The X axis represents the age (BP), while the fill values represent the haplogroups abundance. The most recent dogs here considered are from Late Antiquity (Table S1).

An analysis on the occurrence of dog haplogroups in ancient Eurasian canids (Figure 2 and Table S1) attested the presence of Hg A in canids dated >15,000 BP from Italy and the appearance of Hg D at around ~8000 BP.

The seven haplotypes obtained were checked against the GenBank database, along with sequences from other ancient Italian specimens, which corresponded to seven haplotypes [13,31]. We found that 9 out of 14 haplotypes from ancient Italian specimens matched published samples in the GenBank database (Table 2). In particular, starting from the Late Pleistocene, the sample OWW9, for which Ciucani and colleagues [13] previously detected a match with modern dogs (*n* = 93), also matches ancient interesting specimens. Indeed, the same haplotype was retrieved from a Bulgarian Chalcolithic dog [6] and two historical sledge dogs from the North American Arctic [67]. The other Italian canid (OWW4), which shared the same 57 bp fragment with the sample OWW9, matched with a Mesolithic dog, two Chalcolithic dogs from Bulgaria and Iberia [6,33] and the most recent dogs (Roman dogs from Iberia and historical sledge dogs from North American Arctic) [67,81]. The sample CAF18.0066 matched only with six Late Pleistocene wolves, of which five were from the same site [13] and one from Belgium [48].

In the Late Glacial period, the sample PIC1 showed the occurrence of the haplogroup C, in agreement with the results of coeval ancient dog specimens from Central Europe (Germany-Switzerland) [29] and Italy [34]. PIC1 and the subsequent early Holocene PIC2 specimen showed no similarity with any samples reported in the literature. Moreover, the PIC2 sample attested the presence of haplogroup B in Italy in the early Holocene. These two samples, along with the PIC3 specimen were not confidently assigned to either proto-dogs or wolves, using both morphological and genetic analyses [31]. PIC3 shared the haplotype of one Neolithic Bulgarian dog and four historical sledge dogs from North American Arctic, in addition to more than a hundred modern dogs.

In the Chalcolithic and Bronze Age, our samples were assigned to haplogroups C, D (in the sole IPC6 sample) and A. Four samples (IPC1, IPC8, IPC6 and FA8) matched no other sequences in the GenBank database. Moreover, PIC4 and ORD7 matched only modern dog samples, and in particular ORD7 matched with a modern Bulgarian Hound dog [82].

Four Bronze Age samples from Solarolo and Cesena (ORD3, ORD14, ORD20 and FA7) showed similarity with Late Pleistocene wolves and modern dogs. Lastly, FA5 and PIC5 matched with recent samples: a Medieval wolf, and historical and modern dogs, respectively (Table 2).

### 3.2. Phylogenetic Analyses

**Figure 3:**
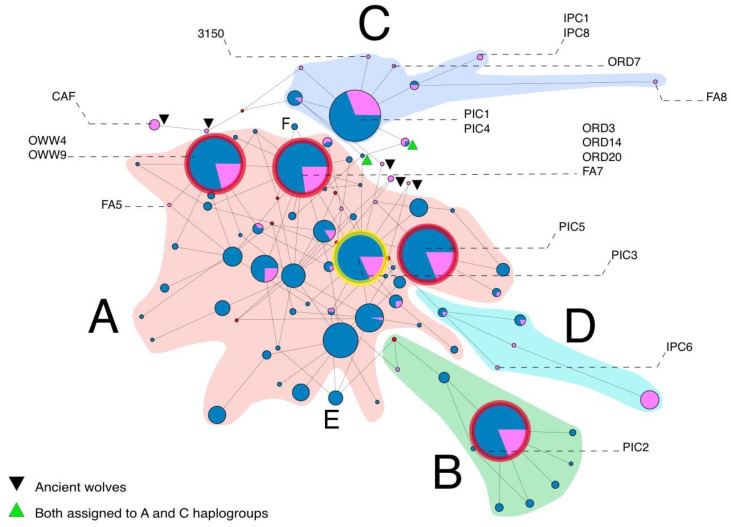
Median Joining network based on the short fragment (57 bp) alignment containing ancient and modern dogs and ancient Italian wolves. European samples are shown in purple, Asian samples in blue. The red rings highlight haplotypes that contain both ancient Italian and Bulgarian samples (alongside modern samples); the yellow ring highlights a haplotype that contains, among others, ancient Italian, Iberian and Bulgarian samples. The four main canine haplogroups are highlighted in pink (Hg A), green (Hg B), purple (Hg C) and light blue (Hg D). Minor haplogroups E and F contain only modern individuals. Red dots represent median vectors. Samples included in the network are listed in the Table S1.

The network (Figure 3) clearly distinguished the four main dog haplogroups (A, B, C and D), despite its construction using the short alignment (57 bp). The ancient Italian samples presented high levels of genetic variability, being scattered across the whole network.

Within Hg A, five samples (ORD3, ORD14, ORD20, FA5 and FA7) are placed only one mutational step away from the two samples of the same haplogroup, OWW4 and OWW9, which were dated, respectively, at ~24,700 and ~20,000 years BP and were recovered from the site of Cava a Filo [13]. A connection is evident between the samples ORD3, ORD14, ORD20 and FA7 and the Italian sample PIC3 from Romanelli Cave published by Verginelli et al. [31], situated one mutational step away.

It is interesting to note the proximity of the ancient Iberian samples sequenced by Pires et al. [33], LYEP44 (~14,000 BP), and the Mesolithic ones (LYEP3, LYEP68B, LYEP74, LYEP75), which fall in the same 57 bp haplotype of the Late Glacial sample PIC3 [31].

Almost all of the ancient Bulgarian specimens published by Yankova et al. [6] shared haplotypes with ancient Italian samples. In fact, among the samples from Bulgaria, the Neolithic and Chalcolithic samples (Ohod19, Urdo14 and Bal16), and a Late Antiquity sample (KpAn15), shared the same 57 bp haplotype with PIC5. Conversely, a Chalcolithic sample (OkGl10) fell in the same haplotype of OWW9. Two Neolithic samples (MPGra13 and ToPro1) were associated with PIC3, and some Chalcolithic samples (ToPro7 and SulPο9) were associated with ORD3, ORD14, ORD20 and FA7.

The samples belonging to Hg C tell a slightly different story. The Italian samples PIC1 and PIC4 share the same haplotype in particular with a Newgrange sample dated at ~4800 BP [8] and a dog from Germany dated at ~12,500 BP [29]. As mentioned previously, ORD7 shared the same haplotype with a modern sample, a Bulgarian Hound dog, and it is one mutational step away from ancient Italian PIC1 and PIC4 samples. Moreover, a Late Glacial Italian dog from Boschin et al. [34] is also situated one mutational step away from PIC1 and PIC4 samples.

For haplogroup B, we highlighted the proximity of some Neolithic and Chalcolithic Bulgarian samples (Slat11, Burg22 and Doln8), which belonged from the same haplotype of PIC2.

**Figure 4:**
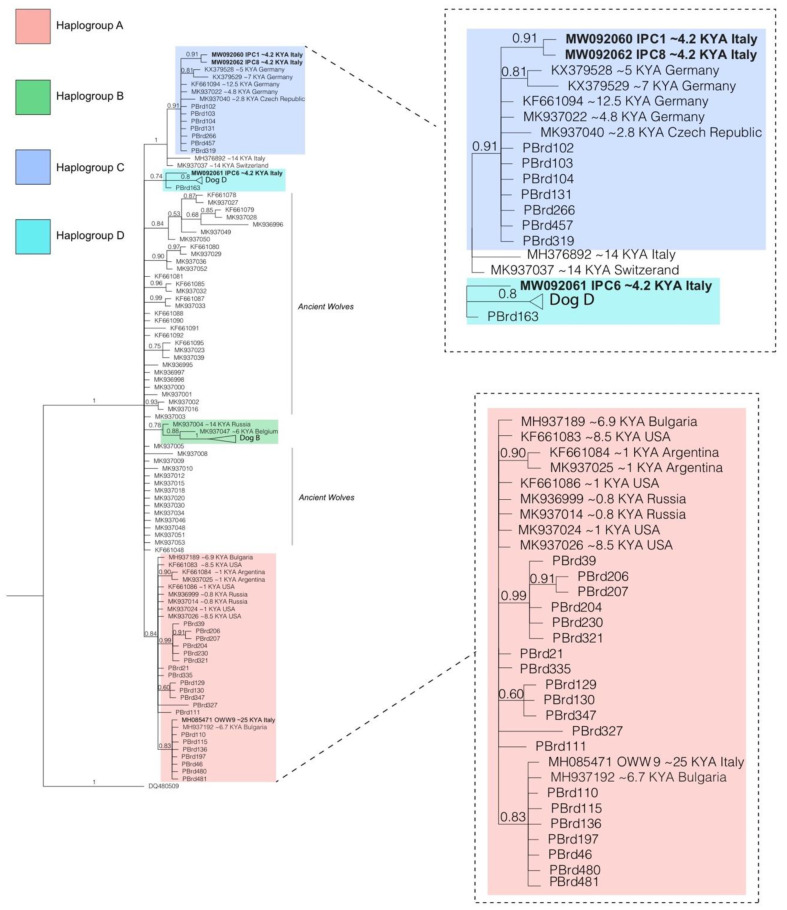
Bayesian tree of ancient and modern dogs and wolves based on the 360 bp database of mtDNA (Table S1). Samples from this study are highlighted in bold. Ancient canid specimens in the three enlargements are labeled with the respective country of origin and their approximate reported age. Monophyletic clusters of modern canid sequences are collapsed and identified by the dog clade to which they belong. PBrd denotes modern dog samples available from literature. The outgroup (a coyote) is not shown. Samples included in the tree are listed in the Table S1.

The Bayesian tree (Figure 4) shows a well resolved mitochondrial phylogeny with a good support of the branches, despite being built from a limited mitochondrial fragment (360 bp). The dogs fall within the main four clades (A–D) and the topology of the tree is similar to those who include large portions of the mitogenome (e.g., [34]). The samples studied herein fell in different canine clades. IPC1 and IPC8 were situated closer to the sister groups constituted by modern dogs belonging to Hg C, near the Neolithic samples CTC and HXH [10] and the dog dated at ~12,500 BP [29], all retrieved in Germany. The GenBank acc. n. MK937022 and MK937037 refer to canids from the Kesserloch cave in Switzerland dated at, respectively, ~11,700 and ~14,100 BP [66]. The first immediately branches off the dog Hg C and the second falls inside the variability of this haplogroup. The specimen MK937040 is a canid from Predmosti in the Czech Republic, dated at ~2900 BP [27,66]. Additionally, the sample highlighted as the first dog specimen from Italy [34] could probably not be correctly placed in this tree as in the one made by the authors considering the whole mitogenome because of the presence of several unassigned bases (N) for this sample in the 360 bp region here considered. Moreover, the Italian sample IPC6 branches off immediately before the clade of the modern dogs of the Hg D. Interestingly, the Late Pleistocene sample OWW9 was placed inside the branch of many modern dog breeds with Hg A, along with two Bulgarian ancient dogs (GenBank acc. n. MH937189 and MH937192) dated, respectively, to the Neolithic and the Chalcolithic age [6] and some New World dogs.

## 4. Discussion

As highlighted in previous studies, the analysis of southern [13,31,33] and southeastern [6] European canids is vital to our understanding of dog migration, admixture and replacements in the context of domestication. This is particularly relevant as the three glacial refugia, the Italian, Iberian and Balkan peninsulas, may have played an important role in the first population dynamics of ancient dogs.

Few ancient Italian canid samples have been genetically analyzed thus far, and they are mainly composed of wolves [12,13,31,34,35]. To date, only three studies, represented by four samples unambiguously morphologically and genetically attributable to dogs, attempted to investigate the genetic variability of ancient dogs from the Italian Peninsula [5,31,34].

With this study, we have enhanced the understanding of the mitochondrial variability of ancient dogs in Italy. Despite the limited number of samples analyzed herein, this research provided a preliminary overview of the population patterns and variability in ancient Italian dogs. In particular, this study was focused on the period between ~24,700 and ~3000 years BP, in an area where previous findings highlighted the presence of Late Pleistocene canids carrying dog Hg A [13].

Dogs are conventionally grouped in four main clades (A–D) [54,68]. Frantz et al. [9] highlighted that, based on the available data, ancient European dogs belonged to Hg C or D, with the Hg C as the most frequently observed haplogroup in Europe prior to the Neolithic. However, most modern European dogs belong to Hg A and Hg B. Frantz et al. [9] suggested that their findings were due to a partial replacement by East Asian dogs, instead of the result of genetic drift alone. According to Ollivier and colleagues [36], the first haplogroup present in Europe prior to the Neolithic was haplogroup C, while haplogroups A and D are thought to have arrived in Europe during Neolithic and post-Neolithic migrations alongside humans, i.e., from the Near East for Hg D, and successive Pontic steppe migrations during the Bronze Age for Hg A [36]. Recently, thanks to the analysis of 27 new ancient dog genomes, Bergstrom et al. [5] detected the presence of Hg A in a Mesolithic Karelian dog from northern Russia (dated at 10,930 years BP), and in a Neolithic Croatian sample (dated at ~4500 years BP). They also found that a single Neolithic Hg A dog from a Megalithic site in Sweden, dated at 5000 BP, can be modeled as a single-source proxy for 90 to 100% of the ancestry of most European dogs [5].

Through our phylogenetic analysis, we evidenced a great degree of variability between 11 ancient Italian samples, where samples were grouped in seven different haplotypes of the haplogroups A, C and D. Only a single sample (CAF18.0066), likely a wolf, fell within the wolf Hg 2 and matched with six Late Pleistocene wolves, of which five were from the same site [13], and one was from Belgium [48]. It is important to note that the Late Pleistocene Italian samples included in the analyses (OWW9, OWW4 and CAF18.0066) have not yet been morphometrically analyzed; thus, no conclusion can be drawn about their possible placement as Paleolithic dogs.

Despite that the first dog populations in Europe were perceived to belong to haplogroup C [36], the presence of a ~24,700-year-old Hg A specimen (OWW9) from Italy triggered the need to further investigate European domestication origins [13]. According to the network analysis, five of the samples from this study (FA5, FA7, ORD3, ORD14 and ORD20) appear to be related to OWW9, as they are positioned only one mutational step away from it. This further supports the early presence of Hg A in Europe.

It is worthy to note the diachronic genetic continuity of the peculiar haplotype carried by the Late Pleistocene OWW9 specimen, as highlighted by the matches in the GenBank database. The same haplotype was present in a Chalcolithic dog from Bulgaria [6], in two historical sledge dogs from the North American Arctic [67], and in ~100 modern dogs (Table 2 and Figure 3).

Our data suggest that there are close relationships between Italian, Iberian [33] and Bulgarian ancient specimens [6]. Several samples shared the same haplotype or were placed few mutational steps from the Italian samples studied herein. Moreover, most of these dated from the Late Pleistocene to the Bronze Age, belong to the Hg A. This sheds light on the history of this lineage, on the population dynamics of dogs in these areas, and in the broad context of dog domestication.

The current study, although based on a limited number of samples and focused on a small portion of the mtDNA genome, highlights the importance of ancient Italian samples to the understanding of past population dynamics and mtDNA variability in dogs. However, since mtDNA has a limited resolution, particularly in this case since a limited fragment was amplified, future studies will certainly benefit from the application of next-generation sequencing (NGS) technologies to analyze entire mito- or nuclear-genomes of ancient canids.

## 5. Conclusions

Our results on the close proximity between Italian samples and those from the Iberian [33] and the Balkan [6] Peninsulas, combined with the evidence published by Pires and colleagues [33], which suggested the possibility of a pre-Neolithic local domestication processes from Iberian wolves, suggest that these areas are of particular interest for domestication dynamics. The acquisition of a broader set of ancient samples from these areas, including ancient representatives from the Late Pleistocene period, is required to further clarify the details of dog domestication and evolution. We highlight that any study that intends to analyze ancient dogs for the analysis of domestication will certainly have to be based on all available evidence from archaeology, morphology and genetics.

## Figures and Tables

**Table 1:** Recovery location, dating and morphological identification of the selected samples.

Sample	Archaeological Site	Dating (Years BP) ^1^	Species
CAF18.0066 *	Cava a Filo, Bologna	25,005–23,842	Wolf
ORD1	Via Ordiere, Solarolo	3600–3280	Dog
ORD2		3600–3280	Dog
ORD3 *		3600–3280	Dog
ORD6		3600–3120	Dog
ORD7 *		3600–3120	Dog
ORD8		3600–3280	Dog
ORD9		3600–3280	Dog
ORD13		3600–3280	Dog
ORD14 *		3580–3380	Dog
ORD15		3600–3280	Dog
ORD16		3600–3280	Dog
ORD20 *		3600–3280	Dog
NIP10	Celletta dei Passeri, Forlì	5350–4250	Dog
FA1	Foro Annonario, Cesena	3400–3120	Dog
FA5 *		3290–3120	Dog
FA7 *		3290–3120	Dog
FA8 *		3290–3120	Dog
FA9		3290–3120	Dog
FA10		3290–3120	Dog
IPC1 *	Ipercoop, Riccione	4750–3600	Dog
IPC6 *		4750–3600	Dog
IPC7		4750–3600	Dog
IPC8 *		4750–3600	Dog
CVGS2	Centro VGS, Cattolica	4250–3600	Dog
CVGS3		4250–3600	Wolf
CVGS6		4250–3600	Dog

^1^ All samples were dated through a relative chronology except for CAF18.0066, whose dating is a result of accelerator mass spectrometry (AMS) dating of other samples from the same stratigraphic unit (S.U.). The conversion of the relative dating from BC to BP was done by the addition of 1950 years. * Highlights the samples for which PCR amplifications were successful.

**Table 2:** Matches of ancient Italian samples sequences against the GenBank database.

Sample ID	Ref.	Dating (Years BP)	Length (bp)	mtDNA Hg	Matches in GenBank
OWW9	[13]	Late Pleistocene (~24,700)	360	A	1 Chalcolithic dog: MH937192 [6]; 2 historical dogs: LR742870, LR742752 [66]; 93 modern dogs
OWW4	[13]	Late Pleistocene (~20,000)	57	A	1 Late Pleistocene canid MH085471; 1 Mesolithic dog: LM993795 [83]; 2 Chalcolithic dogs: MH937192 [6], KY014656 [33]; 2 Roman dogs: KY014674, KY014672 [81]; 2 historical dogs: LR742870, LR742752 [67]
CAF18.0066	This study	Late Pleistocene (~24,400)	57	2 (Wolf Hg)	6 Late Pleistocene wolves: MH085470, MH085472, MH085474, MH085475, MH085476 [13], DQ852650 [48]
PIC1	[31]	Late Glacial (~14,600)	257	C	/
PIC2	[31]	Early Holocene (~9800)	257	B	/
PIC3	[31]	Early Holocene (~9600)	257	A	1 Neolithic dog: MH937189 [6]; 4 historical dogs: LR742837, LR742829, LR742796, LR742751 [67]; >100 modern dogs
IPC1	This study	Chalcolithic/ Bronze Age (~4200)	360	C	/
IPC8					
IPC6	This study	Chalcolithic/ Bronze Age (~4200)	360	D	/
PIC4	[31]	Chacolithic (~4100)	257	C	~ 60 modern dogs
FA7	This study	Bronze Age (~3400)	57	A	3 Late Pleistocene wolves: MK937053, MK937034, MK937030 [65]; 1 Neolithic dog: MH937190 [6]; 1 Chalcolithic dog: MH937193 [6]; 5 Chalcolithic dogs from China: MN699630, MN699627, MN699629, MN699618, MN699610 [60]; 7 historical dogs: LR742848, LR742809, LR742793, LR742763, LR742760, LR742748, LR742742 [67]; 2 New World ancient dogs: MK937024, MK937026 [29]; 1 historical wolf: MH559227 [12]; >100 modern dogs
ORD3					
ORD14					
ORD20					
ORD7	This study	Bronze Age (~3400)	57	C	1 modern dog
FA5	This study	Bronze Age (~3200)	57	A	Medieval wolf: DQ852643 [48]
FA8	This study	Bronze Age (~3200)	57	C	/
PIC5	[31]	Bronze Age (~3000)	262	A	6 historical dogs: LR742834, LR742833, LR742827, LR742826, LR742821, LR742749 [67]; >100 modern dogs

## Supplementary Materials

The following are available online at https://www.mdpi.com/2073-4425/11/12/1409/s1, File S1: Information about the archaeological contexts, Figure S1: Pictures of the specimens from which the samples were collected, Figure S2: UPGMA Tree based on the alignment at 57 bp with Italian samples in bold, and reference specimens with well-defined dog matrilines, Figure S3: UPGMA Tree based on the alignment at 360 bp with Italian samples in bold, and reference specimens with well-defined dog matrilines, Table S1: List of the samples here analyzed and the modern and ancient specimens available in the literature, utilized for the network and the Bayesian phylogenetic tree, along with the data from ancient Eurasian dogs used for the graphical representation of past haplogroups distribution in Figure 2. This table also includes the mtDNA variants of the specimens here analyzed with respect to the dog reference used.
