# Supplementary material for: Population Dynamics in Italian Canids between the Late Pleistocene and Bronze Age
Source: Genes (Basel). 2020 Nov 26;11(12):1409. doi: 10.3390/genes11121409 (PMC7761486; doi:10.3390/genes11121409)
Supplement: Supplementary file 1 [file genes-11-01409-s001.zip › Supplementary files/File S1.docx]

**Population dynamics in Italian canids between the Late Pleistocene and Bronze Age**

**Koupadi Kyriaki, Francesco Fontani, Marta Maria Ciucani, Elena Maini, Sara De Fanti, Maurizio Cattani, Antonio Curci, Gabriele Nenzioni, Paolo Reggiani, Adam Jon Andrews, Stefania Sarno, Carla Bini, Susi Pelotti, Romolo Caniglia, Donata Luiselli and Elisabetta Cilli**

**Supplementary Materials**

**The Archaeological Contexts**

*Cava a Filo, Bologna*

Cava a Filo (or ex Cava Filo) is located northeast of Castello Mountain in San Lazzaro di Savena, near Bologna (EPSG:4326 N: 44.442772, E: 11.379824). The site’s name derives from its recent use as a quarry, since it is in an outcrop of chalk. The site was formed with fluvio-karsic galleries in the Late Pleistocene. It is an important reference site for chronological and paleo-environmental reconstructions of the Last Glacial Maximum in Italy (ca. 24,000-18,000 years BP) [[1,2]](https://www.zotero.org/google-docs/?1CKz8P). The site has returned huge faunal remains of species associated with cold climates and open environments. The remains were predominantly *Bison priscus, Canis lupus, Capreolus capreolus* and *Megaloceros giganteus. Canis lupus* was the only carnivore retrieved from the site [[2]](https://www.zotero.org/google-docs/?OdssBH). A phalanx recovered from the stratigraphic unit (S.U.) 99, and morphologically identified as wolf, was selected for palaeogenetic analyses at the ancient DNA Laboratory in Ravenna (Sample ID: CAF18.066). This stratigraphic unit was dated though the analysis of two specimens of *Bison priscus* (GrA-52971: 23,842-24,309 cal BP 2σ and GrN-32576: 23,844-24,387 cal BP 2σ - Groningen lab) [[3]](https://www.zotero.org/google-docs/?7OuNg0) and a *Canis lupus* (sample ID: OWW9) (ETH-82629: 24,407-25,005 cal BP 2σ - Zurich lab) [[4]](https://www.zotero.org/google-docs/?Ai8NiL).

*Via Ordiere, Solarolo*

The settlement of via Ordiere in Solarolo (Ravenna, EPSG:4326 Ν: 44.376227, E: 11.819321) has been extensively studied since 2006. The site was inhabited between the Middle and Late Bronze Age (1650-1170 BC). The stratigraphic relationships and the chrono-typological seriation of ceramic types indicate that the majority of archaeological evidence at Solarolo dates to BM2. The latter three stages (BM3, BR1, BR2) have only been identified by the presence of surface fragments [[5–8]](https://www.zotero.org/google-docs/?eZwTsq). Together with structural features and the material culture (pottery, lithic tools, bone and antler artifacts), a large variety of animal remains have been recovered. A systematic zooarchaeological study of the animals exploited at Solarolo was conducted on a sample of ~8,000 osteological fragments. Domestic animals were predominant, while wild species (deer, hare, foxes, birds, and fishes) were less well represented. Sheep and goats represented almost half of the identified specimens (NISP), followed in abundance by pigs and cattle, while equids and dogs were scarce [[5,9]](https://www.zotero.org/google-docs/?BK6Gnm).

In total, 87 skeletal remains of dogs were recovered, including maxillae, mandibles, teeth, skull bones, atlas’, scapulas’, humerus’, radius’, ulnas, carpals, metacarpals, pelvis’, femurs, tibias, metatarsals, and phalanges. Only fragments of maxillae and mandibles baring teeth (ORD1, ORD2, ORD6, ORD8, ORD9, ORD13, ORD15, ORD16, ORD20) were selected for DNA analysis. Poor DNA preservation in samples from S.U.’s 19, 36 and 3, meant that we had to resample these S.U.’s (ORD3, ORD7, ORD14). We estimated that the final analyzed samples, each from a different S.U., represented nine different individuals. One sample was identified as a juvenile, four as adults, while the age of the remaining four was not determinable. Morphological analysis with a general evaluation of the growth state of bones, revealed that all individuals were of a similar size, mean height = 43 cm, the average height of Bronze Age dogs in Northern Italy [[10]](https://www.zotero.org/google-docs/?umw6Kv).

*Celletta dei* *Passeri*, *Forlì*

The ancient necropolis “Celletta dei Passeri” is a Chalcolithic site recovered via a rescue excavation (EPSG:4326 Ν: 44.2227, E: 12.0407). More than seventy tombs were found during the excavation of the site. Axes, flints, and animal remains were recovered in addition to human remains [[11]](https://www.zotero.org/google-docs/?iuUOvY). A tooth analyzed in this study was found in Tomb 14, which was one of nineteen teeth attributed to a juvenile *Canis familiaris*.

*Foro Annonario, Cesena*

The Foro Annonario site was discovered during constructions in the main square of Cesena (EPSG:4326 Ν: 44.136235, E: 12.242511). Layers were dated to the Bronze Age (ΒΜ and BR) or Iron Age [[12]](https://www.zotero.org/google-docs/?TGBcAn). Some areas presented complex stratigraphic sequences indicating a number of settlement phases, evidenced by traces of structures, ceramics, and tools made from animal hard-parts i.e. deer antler and ribs of large herbivores [[5]](https://www.zotero.org/google-docs/?lgPuxO).

During the excavations of the Bronze Age layers, a total of 2,372 animal remains were recovered, which were mostly well preserved and able to be identified to species level. Here, domesticated species were more abundant than wild animals. The small number of wild animal remains were attributed to deer, wild boar, roe deer, fox, European badger, lagomorphs, and birds of the *Anser* genus. The most abundant domestic animals were pigs, followed by sheep /goats, cattle, horses, and dogs. A total of twenty dog remains were recovered [[13]](https://www.zotero.org/google-docs/?5VY1vk), of which nine were available for analysis including six jaw bones (mandibles and maxillas). Teeth (FA1, FA5, FA7-FA10) with roots protected by bone were selected for analysis.

*IperCoop, Riccione*

The pluristratified site of IperCoop-Riccione was discovered via the construction of a supermarket (EPSG:4326 Ν: 43.995933, E: 11.379821). Residential areas and workshop areas with remains of clay and sandstone processing were identified in different levels dated to the Middle and Late Bronze Age [[5,14]](https://www.zotero.org/google-docs/?LynrOq).

The 193 osteological remains recovered included skeletal remains of cattle, sheep/goats, pigs, and a small number of horse and dog bones. Several other bones were identified as deer, wild boar, or birds. The remains of four *Canis familiaris* individuals were found with human bone fragments in a burial located in the south area of the site and dated to the Chalcolithic and Bronze Age transition, according to the stratigraphic sequences [[14]](https://www.zotero.org/google-docs/?ZQPsth). Two of the dogs were adults, and two were juveniles. Teeth of the juveniles exhibited less wear than the adults and a smaller size. They were estimated to have been no more than four months old [[15]](https://www.zotero.org/google-docs/?kCbqvj).

A large variety of skeletal dog remains were available for aDNA analysis. In order to avoid sampling the same individual twice, three petrous bones on the same side of the temporal bone were selected for analysis (IPC6,7,8). A fourth individual was represented by a tooth sample (IPC1).

*Centro VGS, Cattolica*

This settlement located in via Cabrigniola, Cattolica was also discovered during rescue excavations (EPSG:4326 N: 43.956808, E: 12.73698). The stratigraphy and the chrono-typological seriation of ceramic types indicate a continuous occupation of the site spanning the Early Middle Bronze Age and the first phase of the Middle Bronze Age (2300-1550 BC) [[16]](https://www.zotero.org/google-docs/?XOcKRj).

In addition to ceramics, lithic tools, and traces of different types of buildings, a large amount of animal remains was found [3]. A significant number of these bones were not identified due to their poor preservation states, though those identified were mostly domesticated species. Pigs and sheep/goats were predominant, while cattle remains were scarce. Canids remains were few, 12 in total, belonging to at least three different individuals. Three specimens were selected for the analysis herein, two of which were identified as dogs (CVGS2, 6), and one as wolf (CVGS3) [[17]](https://www.zotero.org/google-docs/?WcHjTJ).

*References*

[1. Ciucani, M.M.; Palumbo, D.; Galaverni, M.; Serventi, P.; Fabbri, E.; Ravegnini, G.; Angelini, S.; Maini, E.; Persico, D.; Caniglia, R.; et al. Old wild wolves: ancient DNA survey unveils population dynamics in Late Pleistocene and Holocene Italian remains. *PeerJ 7:e6424* **2019**, doi:107717/peerj.6424..](https://www.zotero.org/google-docs/?7X4svt)

[2. Paronuzzi, P.; Berto, C.; Ghezzo, E.; Thun Hohenstein, U.; Massarenti, A.; Reggiani, P. Nota preliminare sulla sequenza UMG di ex Cava a Filo (Croara, BO): gli aspetti stratigraficosedimentari, paleontologici e antropici alla luce delle ultime indagini (2006–2016). *Memorie dell’Istituto Italiano di Speleologia* **2018**, *2*, 131–144.](https://www.zotero.org/google-docs/?7X4svt)

[3. Paronuzzi, P.; Breda, M.; Ghezzo, E.; Reggiani, P. La fauna tardo-pleistocenica a macromammiferi del sito di ex Cava a Filo (indagini 2006-2011): tassonomia e quadro cronologico-paleoambientale.](https://www.zotero.org/google-docs/?7X4svt)

[4. Ciucani, M.M.; Palumbo, D.; Galaverni, M.; Serventi, P.; Fabbri, E.; Ravegnini, G.; Angelini, S.; Maini, E.; Persico, D.; Caniglia, R.; et al. Old wild wolves: ancient DNA survey unveils population dynamics in Late Pleistocene and Holocene Italian remains. *PeerJ 7:e6424* **2019**, doi:107717/peerj.6424.](https://www.zotero.org/google-docs/?7X4svt)

[5. Maini, E. LO SVILUPPO DELL’ALLEVAMENTO IN EMILIA-ROMAGNA: Aspetti economici e implicazioni sociali nella gestione della risorsa animale durante l’età del Bronzo. PhD Thesis, University of Bologna, 2012.](https://www.zotero.org/google-docs/?7X4svt)

[6. Cattani, M. Gli scavi dell’abitato dell’età del Bronzo di via Ordiere. *IpoTESI di Preistoria* **2009**, *2*, 115–130.](https://www.zotero.org/google-docs/?7X4svt)

[7. Cattani, M.; Miari, M. La Romagna tra antica e recente età del Bronzo.; Modena, 2010.](https://www.zotero.org/google-docs/?7X4svt)

[8. Cattani, M. Gli scavi nell’abitato di via Ordiere a Solarolo (RA) e il progetto di ricerca sull’età del Bronzo in Romagna. *IpoTESI di Preistoria* **2009**, *2*, 115–130.](https://www.zotero.org/google-docs/?7X4svt)

[9. Maini, E.; Curci, A. Le analysi archeozoologiche nel sito di Solarolo- via Ordiere. In *Economia e Ambiente nell’ Italia Padana dell’ età del Bronzo: Le indagini bioarcheologiche*; Beni archeologici - conoscenza e tecnologie Quaderno; Edipuglia: Bari, 2013; pp. 295–312.](https://www.zotero.org/google-docs/?7X4svt)

[10. Tagliacozzo, A.; Mazzorin, J.D.G. Morphological and osteological changes in the dog from the Neolithic to the Roman period in Italy.](https://www.zotero.org/google-docs/?7X4svt)

[11. Bertoldi, F.; Miari, M.; Tagliani, L.; Bartoli, F.; Rasia, P.; Bestetti, F. The eneolithic necropolis of Forlì-Celletta dei Passeri: taphonomy, anthropology and paleonutrition. *Journal of Biological Research* **2012**, *85*, 210.](https://www.zotero.org/google-docs/?7X4svt)

[12. Miari, M.; Negrelli, C., Lo scavo del Foro Annonario](https://www.zotero.org/google-docs/?7X4svt) *In* [*Ritmi di Transizione 2. Dal Garampo al Foro Annonario: ricerche archeologiche 2009-2013*; All’Insegna del Giglio, 2016; pp. 11-12..](https://www.zotero.org/google-docs/?7X4svt)

[13. Gasparini, D.; Fortini, E.; Condini, G., Bendetta; Rossi, T.; Pelegrino, O.; Maini, E. *From Garampo to Foro Annonario: 2009-2013 archaeological research*; Transition Rhythms; All’ Insegna del Giglio: Florence, 2016; ISBN 978-88-7814-719-5.](https://www.zotero.org/google-docs/?7X4svt)

[14. Maini, E. Le analysi archaeozoologiche nel sito di Riccione- Iper Coop. In *Economia e Ambiente nell’ Italia Padana dell’ Età del Bronzo: Le indagini bioarcheologiche*; Quaderni del Consiglio Nazionale delle Ricerche e dell’Università del Salento.; Edipuglia srl: Bari, 2013; pp. 317–328.](https://www.zotero.org/google-docs/?7X4svt)

[15. Maini, E. Un giorno da cani. *Sezione di Museologia Scientifica e Naturalistica* **2015**, *11*, 47–50.](https://www.zotero.org/google-docs/?7X4svt)

[16. Miari, M.; Valli, E.; Bazzocchi, M.; Bestetti, F.; Gatto, L.D.; Mazzanti, C.; Padoanello, S.; Tagliani, L. L’insediamento del Bronzo antico di Cattolica (RN). ). Notizie preliminari, *IpoTESI di Preistoria*, *vol. 2*. **2009**, 1, pp. 37-74. http://ipotesidipreistoria.cib.unibo.it.](https://www.zotero.org/google-docs/?7X4svt)

[17. Maini, E. Le analisi archeozoologiche nell sito di Cattolica- Centro VGS (RN). In *Economia e ambiente nell’ Italia Padana dell’ età del Bronzo: Le indagini bioarcheologiche*; beni archeologici - conoscenza e tecnologie Quaderno; Edipuglia: Bari, 2013; pp. 271–292.](https://www.zotero.org/google-docs/?7X4svt)
