# Supplementary figures and images for: Population Dynamics in Italian Canids between the Late Pleistocene and Bronze Age
Source: Genes (Basel). 2020 Nov 26;11(12):1409. doi: 10.3390/genes11121409 (PMC7761486; doi:10.3390/genes11121409)

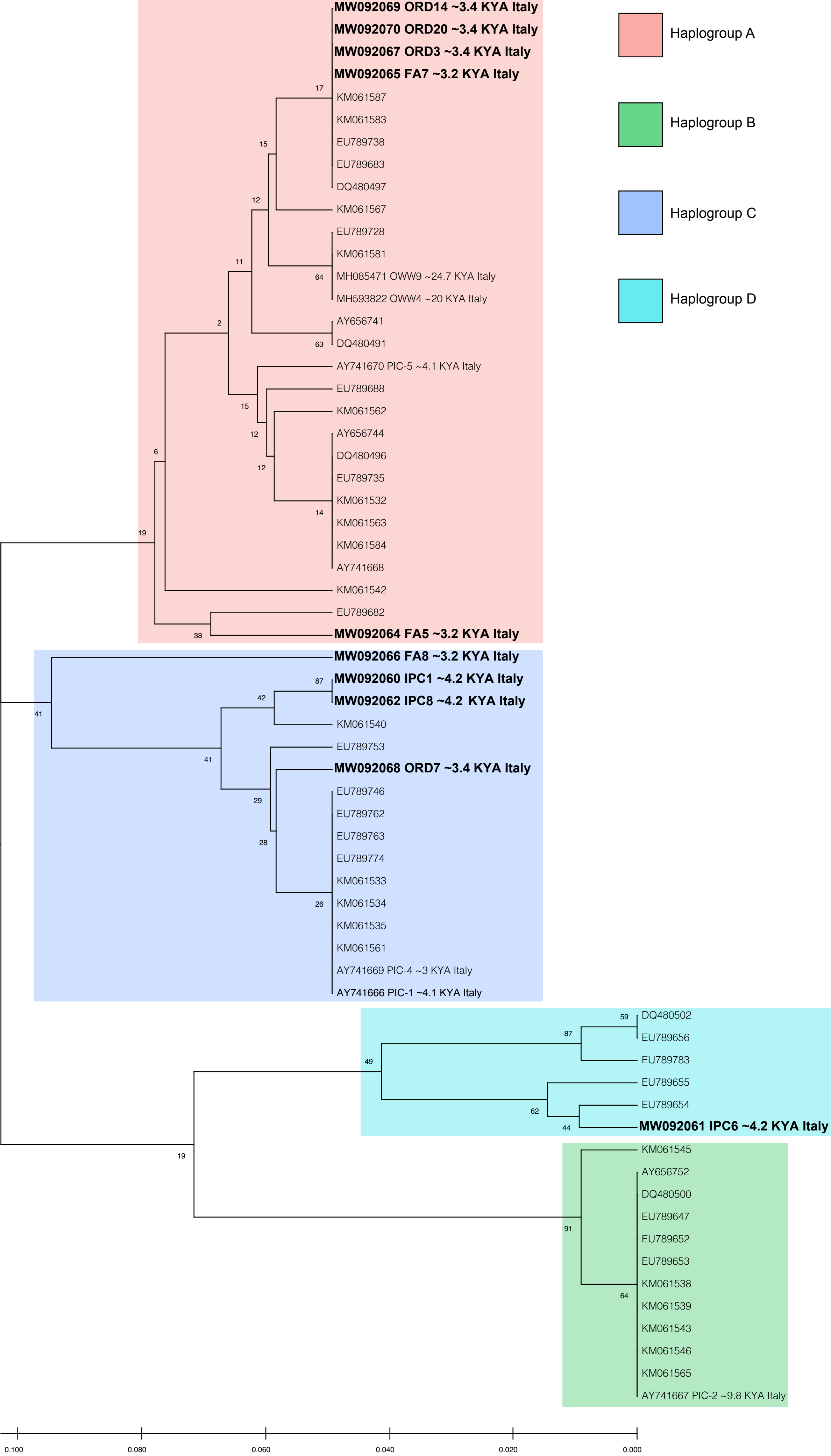

Supplement: Supplementary file 1 [file genes-11-01409-s001.zip › Supplementary files/Fig.S2.pdf]

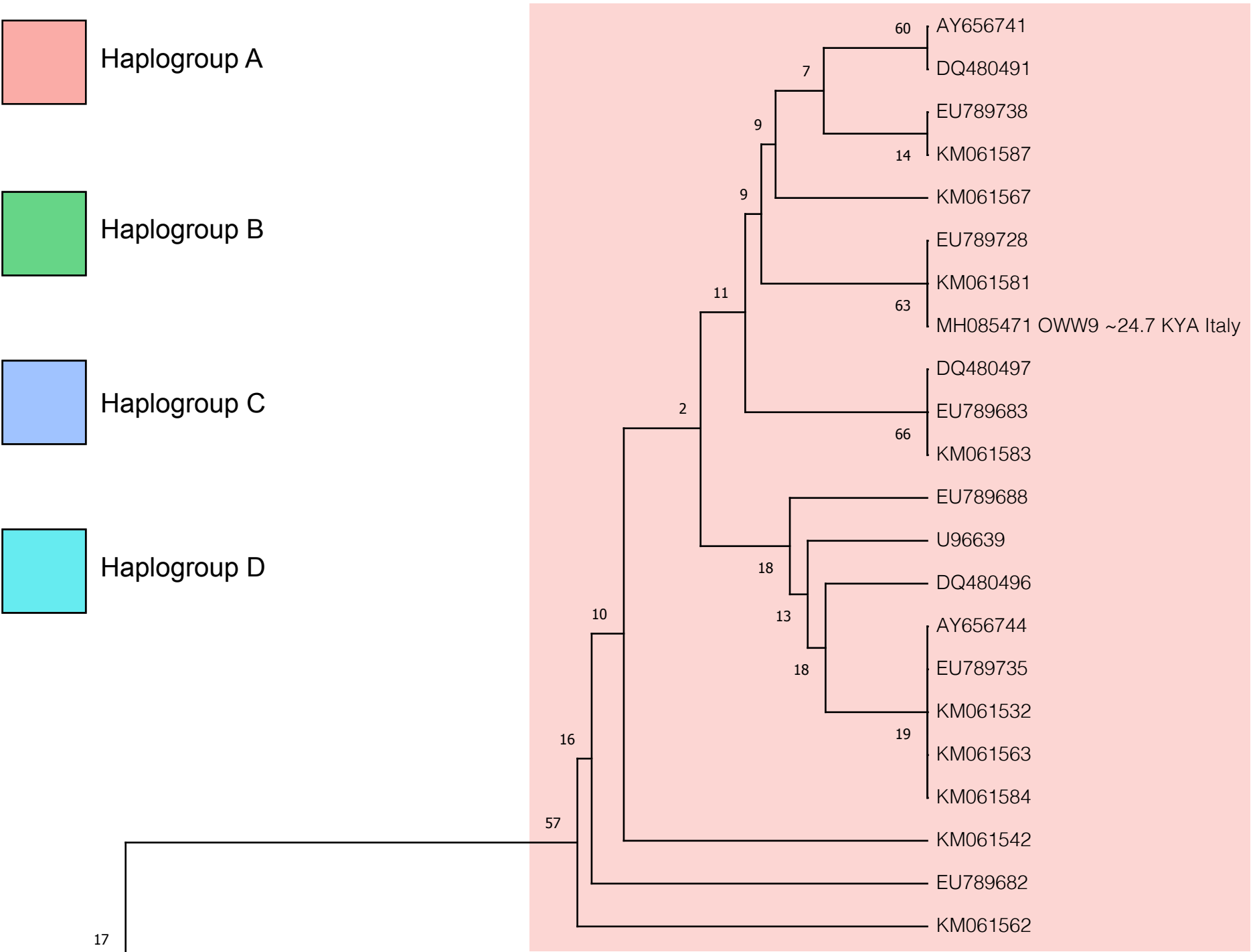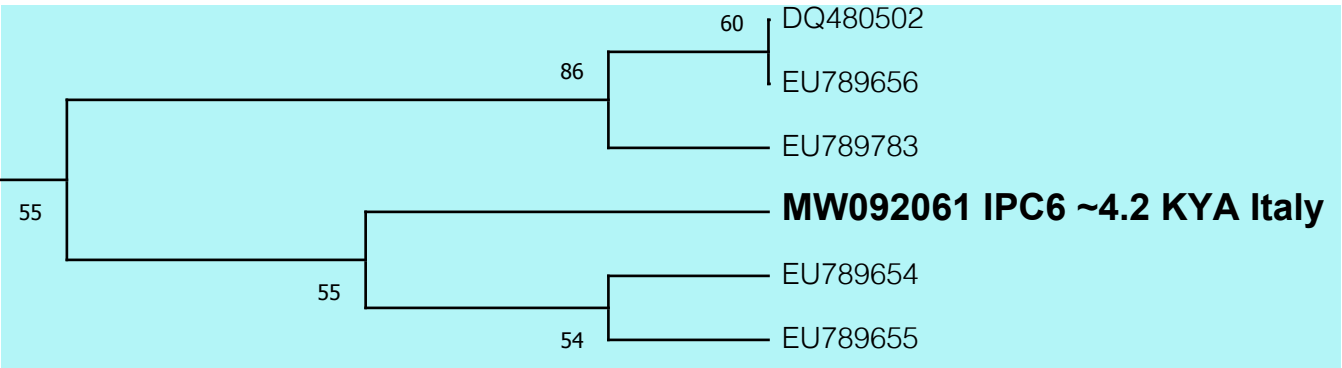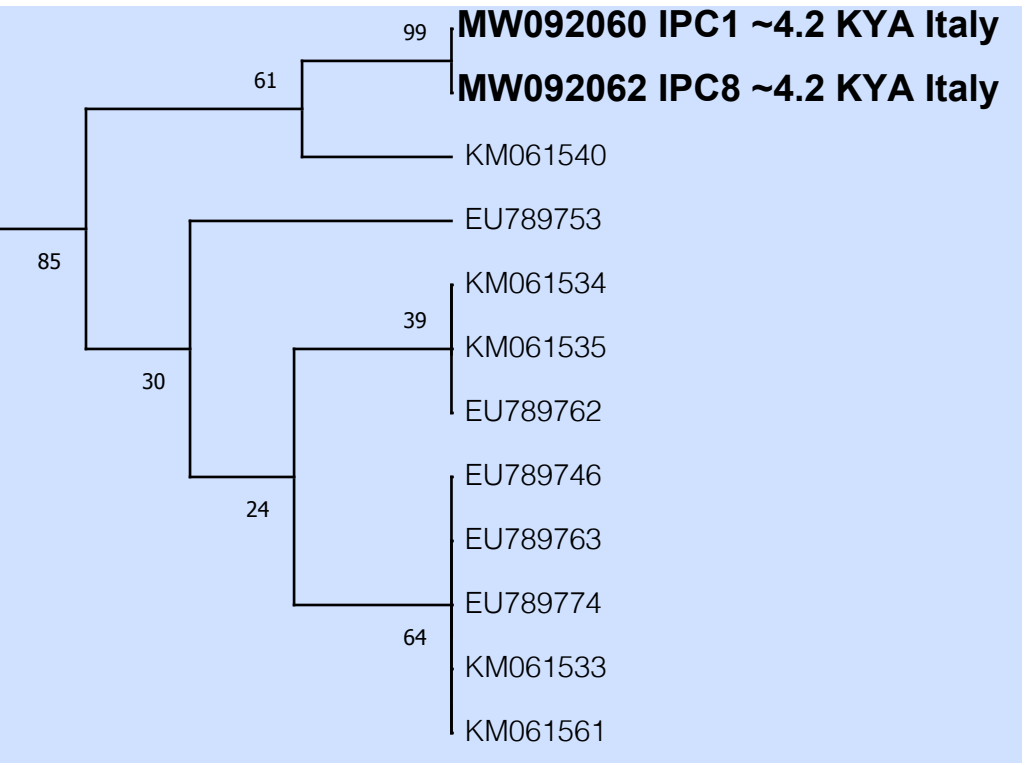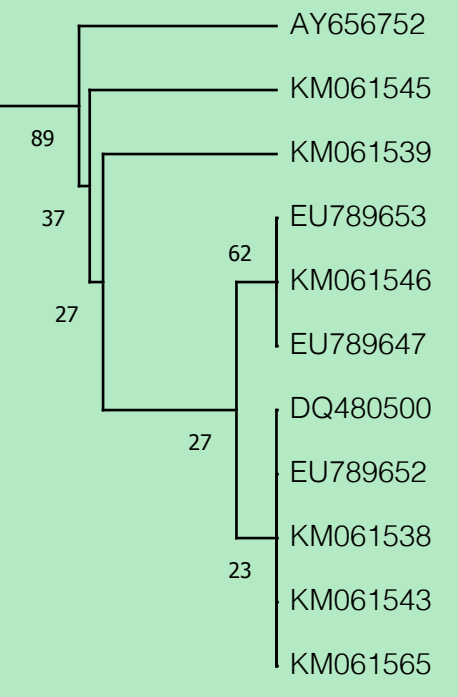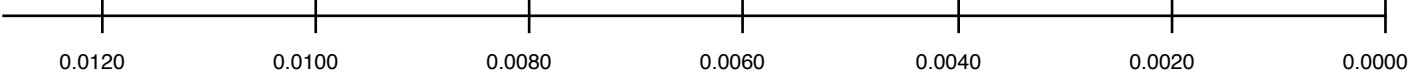

Supplement: Supplementary file 1 [file genes-11-01409-s001.zip › Supplementary files/Fig.S3.pdf]

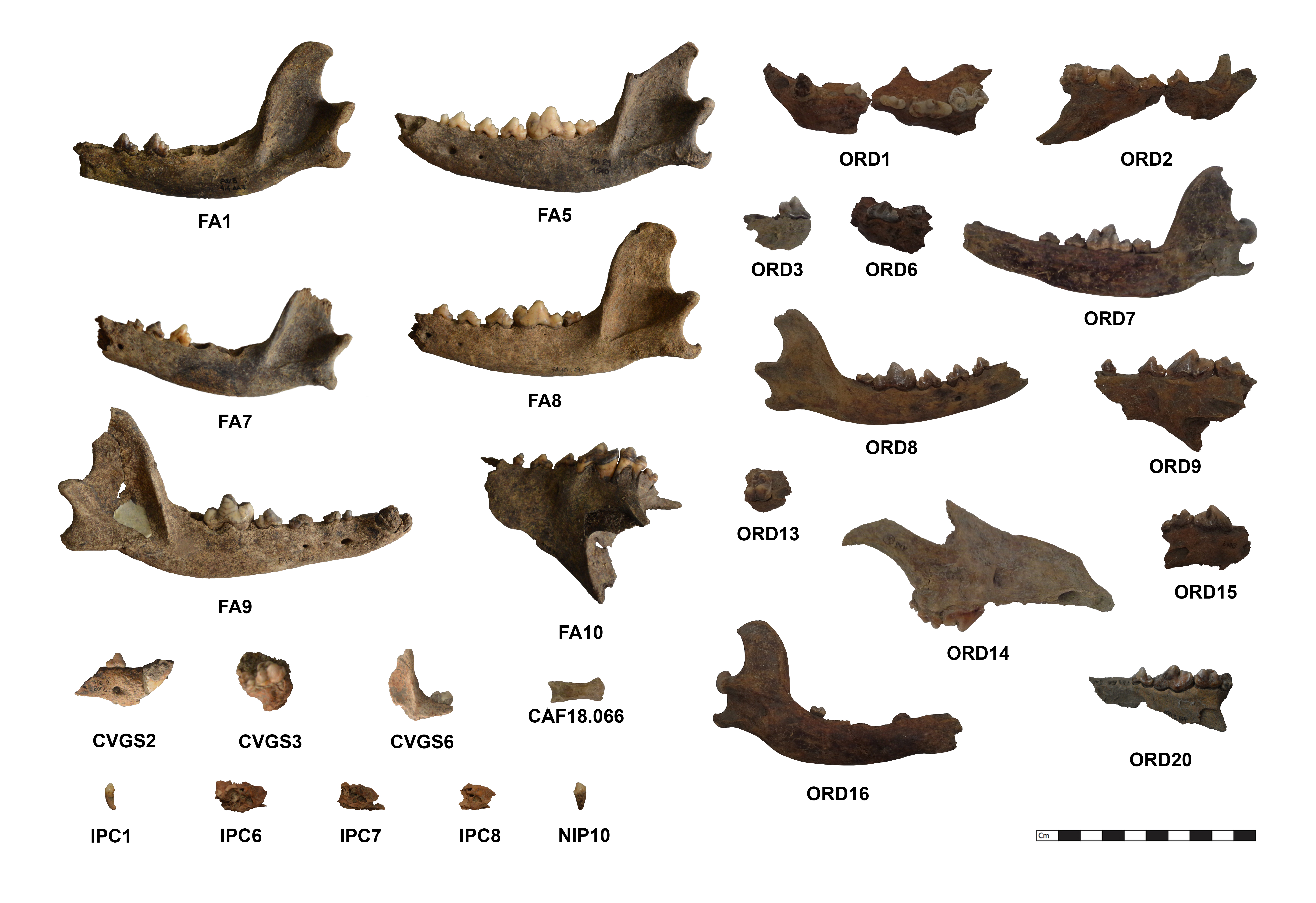

Supplement: Supplementary file 1 [file genes-11-01409-s001.zip › Supplementary files/Figure S1.jpg]
